# Vascular Diseases: Etiologic, Diagnostic, Prognostic, and Therapeutic Research

**DOI:** 10.3390/life13051171

**Published:** 2023-05-12

**Authors:** Dan Mircea Olinic, Agata Stanek

**Affiliations:** 1 First Medical Clinic, “Iuliu Hatieganu” University of Medicine and Pharmacy, 400347 Cluj-Napoca, Romania; 2 Department and Clinic of Internal Medicine, Angiology and Physical Medicine, Faculty of Medical Sciences in Zabrze, Medical University of Silesia, 41-902 Bytom, Poland

The impacts of vascular diseases (VD) include increased morbidity and mortality, which are related to factors such as population aging, socio-economics, or an increasing prevalence of risk factors, e.g., for diabetes, among others. VDs involve peripheral arteries and veins, aortic and visceral abdominal vessels, as well as cervical vessels, such as carotids and vertebral arteries. Cardiac and cerebral complications are often related to vascular diseases. Coronary, carotid, and peripheral arterial conditions have a common atherothrombotic etiology and share many clinical diagnostic, prognostic, and therapeutic interrelations. Additionally, microcirculation is a field of increasing fundamental and clinical research interest, for both arterial and venous diseases. The ongoing COVID-19 pandemic is related to major vascular complications, both on arterial and venous sites. Vascular medicine is not just a field of intense medical research but has also become a distinct medical specialty or subspecialty in many countries.

The clinical research covers specific arterial and venous diseases, their specific impact on organ diseases, as well as the interrelationship among vascular diseases, at multiple sites. New drugs are developed for risk factor control (dyslipidemia, hypertension, diabetes, etc.) or for antiplatelet and anticoagulant therapy, with a significantly beneficial prognostic impact. Revascularization at different arterial sites improves morbidity and mortality. Moreover, interventional procedures on both arterial and venous sites have seen considerable advancement and have been proven to be safe and effective.

This Special Issue collection comprises eight original research articles, three reviews and one brief report, which is summarised below.

Erectile dysfunction (ED), a common male disorder, is often associated with cardiovascular diseases, aging and mental disorders such as depression, while its main cause is vascular. In the paper “The rs2682826 Polymorphism of the NOS1 Gene Is Associated with the Degree of Disability of Erectile Dysfunction”, Perticarrara Ferezin et al. [1] explores whether or not two nitric oxide synthase 1 (NOS1) genetic polymorphisms (rs41279104 and rs2682826) and three PDE5A genetic polymorphisms (rs2389866, rs3733526, and rs13124532) are associated with a risk of ED and the degree of dysfunction if so. This is the first study showing an association between the rs2682826 polymorphism of the NOS1 gene and the International Index of Erectile Function (IIEF) erectile function score (disease severity) in clinical ED patients. The authors showed that for the rs2682826 polymorphism of the NOS1 gene, the CC genotype in clinical ED patients had a positive influence on the values of the IIEF score for erectile function, meaning that carriers of this genotype have less severe erectile function, while carriers of the CT + TT genotype demonstrated a negative association with the IIEF score, implying that the degree of erectile dysfunction amongst these carriers was worse. 

Endothelial dysfunction plays a key role in all vascular disorders [2]. It has been shown that the endothelial function of the femoral artery (FA) may be a promising biomarker of lower-extremity vascular health for peripheral artery disease (PAD) prevention and treatment. In their paper, M. Bapir et al. [3] demonstrated that flow-mediated dilation (FMD) measurements can be performed reproducibly in the common FA using both thigh and calf occlusions, with calf occlusion being better tolerated. The FA-FMD decrease is inversely correlated with age to a similar degree as is FMD in the brachial artery (BA-FMD) in healthy individuals. Wall shear stress (WSS) is lower in the FA on average compared to the BA and decreases to a critical level with age more frequently in the FA than in the BA. The authors concluded that endothelial function declines similarly with age in the FA and the BA in healthy adults. This age-dependent FA enlargement results in a critical decrease in WSS that may explain part of the age-dependent predisposition to PAD.

In addition, increases in carotid intima-media thickness (CIMT) and coronary artery calcification (CAC) are features of subclinical atherosclerosis that might be determined using the genetic background of patients. Among the multiple risk factors, the proprotein convertase subtilisin kexin type 9 (PCSK9) has a great impact on atheroma development. In the paper “Increased Carotid Intima-Media Thickness in Asymptomatic Individuals Is Associated with the *PCSK9* (rs2149041) Gene Polymorphism in the Mexican Mestizo Population: Results of the GEA Cohort”, R. Posadas-Sánchez et al. [4] found that the *PCSK9* (rs2149041) polymorphism is associated with a higher risk of increased CIMT in asymptomatic individuals without coronary artery disease, as determined by the absence of a CAC score. These findings may be used as markers of the risk of CAD events.

One of the vascular diseases which is connected with high mortality and a high risk of limb loss is acute limb ischemia (ALI). In this disease, 30-day mortality and amputation rates are between 10% and 15% [5]. E.M. Arbănași et al. [6], in their observational, analytical, and retrospective cohort study with a longitudinal follow-up, showed the relevance of preoperative inflammatory biomarkers including the neutrophil-to-lymphocyte ratio and platelet-to-lymphocyte ratio in predicting the poor 30-day prognosis of patients with grade II and III ALI, as outlined by the Rutherford classification. The authors concluded that the values of these biomarkers can help raise clinical awareness of the necessity to actively look for initial signs of organ insufficiency.

In the review paper “Bleeding Risk in Patients with Peripheral Arterial Disease”, A. Visona et al. [7] emphasize that the bleeding risk in PAD patients is mutable and should be reconsidered frequently alongside an introduction of associated anticoagulants and antiplatelet treatments. In their review, the authors present several risk scores which improve everyday decision-making.

In another paper, M. Farooqui et al. [8] the evaluated patterns of care amongst patients with strokes caused by acute basilar artery occlusions. The majority of the patients were treated at an urban academic center, which also performed most of the endovascular therapy procedures. Rural hospitals transferred most of the patients to other healthcare facilities. Urban academic hospitals had the highest median length of stay (8.9 days) or the highest cost of hospitalization (USD 117,261), but also the highest rate of home discharge home (32.6%).

In their report, S.J. Wu et al. [9] present a case of prone positioning (PP) being used successfully to treat microscopic polyangiitis-related diffuse alveolar hemorrhage (DAH) and severe acute respiratory distress syndrome (ARDS). The Authors suggest that PP may have a positive effect in terms of improving oxygenation and is superior to extracorporeal membrane oxygenation (ECMO) in the management of DAH-associated ARDS for reducing the risk of mortality and complications of bleeding.

The scientific literature data show that atherosclerosis and pulmonary embolisms (PEs) affect cardiovascular mortality substantially. In their paper, K. Keller et al. [10] investigated the impact of atherosclerosis on the outcomes of patients with deep venous thrombosis (DVT) and identified the differences in DVT patients with and without PE. Among the DVT patients, 36.3% had symptomatic atherosclerosis, and 40.1% had a concomitant PE. DVT patients with symptomatic atherosclerosis were older, more often male, had a higher prevalence of classical cardiovascular risk factors and a higher Charlson comorbidity index. Symptomatic atherosclerosis was associated with increased mortality and hospitalization and worse primary long-term outcomes, as determined via a 2-year follow-up period undergone by DVT patients. DVT patients without a PE had diabetes mellitus and symptomatic atherosclerosis more often compared to DVT patients with a PE, and symptomatic atherosclerosis was associated with isolated DVT (without a PE). The authors concluded that in the case of DVT or PEs, patients should be screened for concomitant atherosclerotic disease.

In another paper, J. Klen et al. [11] estimated the effectiveness of employing perioperative thromboprophylaxis in elective abdominal surgery based on the Caprini or the Padua score. During the single-center prospective observational study, the authors showed that the Caprini score may be better suited than the Padua score is for VTE assessment in surgical patients. Although there were no significant differences in adverse outcomes between abdominal surgical patients who received perioperative thromboprophylaxis based on either the Caprini or the Padua risk scores, a discordant Padua score was noted in almost 40% of patients who had a high Caprini score.

A new classification for primary brainstem hemorrhage (PBH), the ZJUSAH classification, is presented in the paper [12] by J. Zhou et al. This classification allows the prediction of the outcomes of 90-day consciousness of the most extensive (type 3) hematoma based on its volume. Additionally, this model al allows the assessment of 30-day survival outcomes when conservative treatment is applied. The authors underline that this CT-based hematoma classification system has the potential to enable more precise treatment selection and prognostic prediction for PBH patients.

Diabetic retinopathy (DR) is among the several serious microvascular associations associated with diabetes and is a leading cause of blindness; therefore, early detection and timely treatment are essential in order to prevent vision loss in these patients. In their paper, R. Srinivasan et al. [13] demonstrated that artificial intelligence may be useful in DR and that the deep learning algorithm performs better than human graders do, irrespective of whether the images taken from fundus cameras of different specifications are gradable or ungradable images.

In their paper, A. Achim et al. [14] highlight the usefulness of ultrasound in accessing the distal radial artery at the anatomical snuffbox for cardiovascular interventions. Although the role of ultrasound remains optional in the proximal (conventional) radial approach, the authors encourage the use of ultrasound because the artery differs in caliber and position. Therefore, imaging can help the operator perform a clean puncture, which is especially significant since repeated punctures are not only painful but also because any amount of hematoma formation leads to the complete compression of the artery and a failure to access it.

The editors of the Special Issue dedicated to vascular diseases strongly believe that the knowledge contained in this issue will further mobilize scientists to improve etiologic, diagnostic, prognostic, and therapeutic research on patients with vascular diseases.
